# Spatial Frequency Integration During Active Perception: Perceptual Hysteresis When an Object Recedes

**DOI:** 10.3389/fpsyg.2012.00462

**Published:** 2012-10-30

**Authors:** Timothy F. Brady, Aude Oliva

**Affiliations:** ^1^Vision Sciences Laboratory, Department of Psychology, Harvard UniversityCambridge, MA, USA; ^2^Computer Science and Artificial Intelligence Laboratory, Massachusetts Institute of TechnologyCambridge, MA, USA

**Keywords:** spatial frequency, hysteresis, hybrid images, perceptual organization

## Abstract

As we move through the world, information about objects moves to different spatial frequencies. How the visual system successfully integrates information across these changes to form a coherent percept is thus an important open question. Here we investigate such integration using hybrid faces, which contain different images in low and high spatial frequencies. Observers judged how similar a hybrid was to each of its component images while walking toward or away from it or having the stimulus moved toward or away from them. We find that when the stimulus is approaching, observers act as if they are integrating across spatial frequency separately at each moment. However, when the stimulus is receding, observers show a perceptual hysteresis effect, holding on to details that are imperceptible in a static stimulus condition. Thus, observers appear to make optimal inferences by sticking with their previous interpretation when losing information but constantly reinterpreting their input when gaining new information.

## Introduction

One of the fundamental problems of vision is *perceptual organization*: how we combine the patches of color or light that fall on the retina and structure them into larger units like surfaces or objects (Kubovy and Pomerantz, 1981; Nakayama et al., 1995; Palmer, 1999). One often overlooked aspect of perceptual organization is that our visual system breaks down images by spatial frequency early on in the visual pathway, and thus this perceptual grouping process must operate at least partly in the spatial frequency domain (Blakemore and Campbell, 1969; Lindeberg, 1994). This raises several important issues about active perception, because as agents move through the world, the percept of an attended object constantly changes in spatial frequency content (for a review, see Sowden and Schyns, 2006). A painting that subtends several degrees of visual angle when you are 10 feet away from it will subtend nearly your entire visual field if you get close enough to it, thus moving all of the information in the painting into lower and lower spatial frequencies as you approach it. Given this movement through the spatial frequency domain, how does the visual system dynamically combine information from different spatial frequencies in order to recognize objects and faces?

The structure of the world and the nature of our movement in it create an important asymmetry in the availability of visual information about particular visual objects. When approaching an object the perceptual system is constantly gaining new spatial frequency information, adding details to the online representation of an object. Conversely, when an object is receding, the object becomes more ambiguous as we lose detailed information that becomes too high in frequency to be perceived (Blakemore and Campbell, 1969; Graham, 1989), leaving the observer with a coarser percept of the object (for an elegant demonstration, see Pelli, 1999). While we also gain some very low spatial frequency information as we move away from an object, this gain is minimal and moving further from an object results almost entirely in losing high spatial frequency information with little corresponding gain of low spatial frequency information (see the Appendix and Loftus and Harley, 2005 for a comprehensive discussion). In the current work we ask whether the visual system makes use of this asymmetry when integrating information from different spatial frequencies over time.

Previous work on active perception as a function of viewing distance (Pelli, 1999; Loftus and Harley, 2004; Smith and Schyns, 2009) or spatial resolution (Bruner and Potter, 1964) provides key information on the processes guiding spatial frequency integration. First, human observers’ contrast sensitivity function (CSF; measured in cycles/degree) is relatively constant across different stimulus distances (Rovamo et al., 1992), and distance and image size manipulation have a direct counterpart in image resolution changes (Loftus and Harley, 2005; Sowden and Schyns, 2006), which suggest a general mechanism of spatial frequency integration that is independent of the format of presentation. Second, moving backward or forward in spatial frequency space changes the resolution at which an image gains or loses its interpretation. For example, in Bruner and Potter (1964) observers were asked to interpret the content of photographs coming slowly in or out of focus. Importantly, observers tended to “hold on” to the full resolution image when it went out of focus (see also Sadr and Sinha, 2004). This has been interpreted as observers making use of information across time in order to come to the best interpretation of the current image (Sadr and Sinha, 2004).

In addition, many researchers have investigated the order in which we process spatial frequencies when we see an object presented briefly. When visual stimuli are shown, observers generally process them in a coarse-to-fine progression, such that observers are first sensitive to the low spatial frequency information and only later make use of the fine details in the high spatial frequencies (Schyns and Oliva, 1994; Bar, 2004). This may allow observers to make use of the low frequencies of an object to make predictions about the objects identity (Bar, 2004; Bar et al., 2006). However, this coarse-to-fine ordering is not inflexible: spatial frequency integration can be quite fast (Kihara and Takeda, 2010) and the task can determine which spatial frequencies are processed first (Schyns and Oliva, 1999).

**Figure 1:**
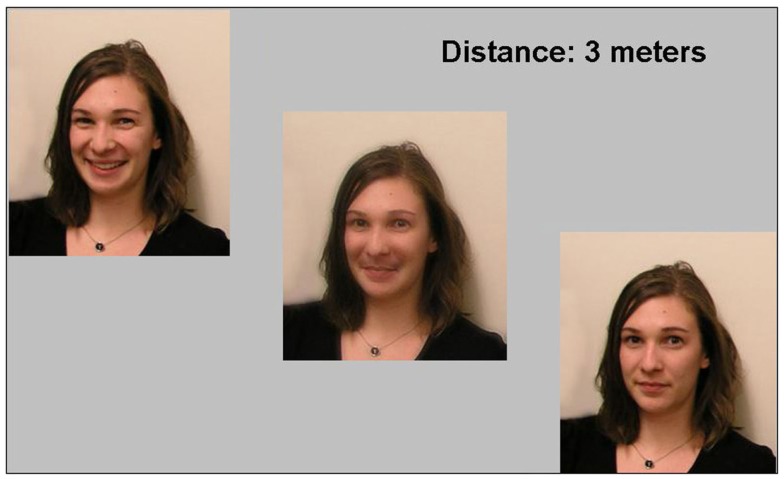
**Example display**. The hybrid image in the middle is composed of the left image (in the low spatial frequencies) and the right image (in the high spatial frequencies). Observers had to stand at the distance indicated and judge whether the hybrid looked more like the left or right component image using a keypad. Because of the human contrast sensitivity function (see Appendix), when viewing this hybrid from close up it should look like the right image; when holding it far away or squinting it should look like the left component image.

Studying the mechanisms of spatial frequency integration is difficult with natural images, since they lose their interpretation when the image gets blurry. However, this question can be addressed with *hybrid* visual stimuli, whose interpretation changes with image resolution. We thus presented observers with hybrid faces – images made up of two different faces, one in the low and one in the high spatial frequencies (see Figure 1; Schyns and Oliva, 1994, 1999; Oliva and Schyns, 1997; Oliva et al., 2006), and probed participant’s perception as they physically moved closer or further from the ambiguous images (change in *physical distance*, Experiment 1); as they examined the images growing or shrinking on a monitor (change in *retinal size*, Experiment 2); or, using hybrid visual scenes rather than hybrid faces (Experiment 3). All three experiments showed a similar and striking interaction in interpreting the same physical input as the percept moved forward or backward in spatial frequency space. Upon approaching, observers acted as though they were independently integrating spatial frequency at each moment. However, when the stimulus was receding, observers showed a *perceptual hysteresis* effect, holding on to details that were imperceptible in a static stimulus condition. These results demonstrate for the first time different kinds of perceptual integration of information during online visual perception: observers tend to stick with their previous interpretation when an image recedes, but constantly reinterpret an image as it approaches.

## Experiments 1 and 2 Materials and Methods

### Observers

Eight observers participated in Experiment 1 and a different set of 18 observers participated in Experiment 2. They had normal or corrected to normal vision and normal spatial frequency perception, as tested with the Functional Acuity Contrast Test (Vision Sciences Research Corporation, San Ramon, CA, USA). All observers gave informed consent.

### Procedure

Each participant saw 18 different hybrid faces (mean RMS contrast 0.24; Peli, 1990; see Figure 1), combining an image of a neutral expression (from one of six different people) with an emotional facial expression of the same person (angry, fearful, happy, or surprised). One face was contained in the low spatial frequencies (Gaussian filter, half-height 30 cycles/image), and one face in the high spatial frequencies (Gaussian filter, half-height 55 cycles/image; see Oliva et al., 2006 and the Supplemental Information for further details). Figure 1 illustrates a trial: the hybrid was always in the center of the display (displayed at 10.9 cm in height), flanked by its two normal component images (with the face representing the high or low spatial frequency components of the hybrid counterbalanced for side). In all experimental conditions, observers were asked to decide whether the hybrid looked more like the left or right face.

In Experiment 1 (effects of *physical distance*), observers (*N* = 8) performed both a *static* and *dynamic* condition, counterbalanced for order across observers. In the *static* condition, we characterized the curve of how observers’ interpretation of hybrids changes as a function of physical distance (from 0.5 to 6 m, by increments of 0.5 m; corresponding to a hybrid size of 0.5–6.2° visual angle). At the beginning of each trial, observers stepped to the distance marked on the screen, and indicated which of the two component images the hybrid looked more like from that current distance using a wireless keypad. The images then disappeared, and a new distance appeared on the screen, indicating where the observer should stand next (distance landmarks were tapped on the floor). After pressing a key, the observer was shown a new trial (as in Figure 1). Over the course of the block, each of the 18 hybrid images was judged from each of the 12 distances, in random order, for a total of 216 trials. In the *dynamic* viewing condition, we examined how perception changes when observers walked toward or away from the image as it was continuously displayed (for previous use of this method, see Pelli, 1999). For each of the 18 hybrid faces, observers started walking forward from the furthest location, or started walking backward from the closest location, and stopped when the hybrid looked equally like both component images (the *point of subjective equality*)^1^. The physical distance at which the observer stood from the screen was recorded. The order of the 36 trials (18 faces, each seen starting at the furthest and the closest distance) was randomized.

Experiment 2 (effect of *retinal* size) mirrored the method of Experiment 1, except that observers (*N* = 18) sat one meter from a 30 inch computer monitor on which hybrids were displayed, ranging in size from 6.2° of visual angle to 0.45°of visual angle – approximately matching the visual angle they subtended at 0.5 and 7 m in the first experiment^2^. While controlling for context effects and extra retinal-information that accompanies real world motion, the retinal size manipulation allowed us to more closely examine the situation where an object is receding from or approaching an observer as she stays in the same location. Stimuli approaching an observer – looming – has known effects on other domains of cognitive processing (Maier et al., 2004) and face processing (Pilz et al., 2011).

A *static* condition (with the same 216 trials as in Experiment 1) was used to characterize the curve that shows how observers’ interpretation of hybrids changes as a function of retinal size. After pressing a key, the three images appeared at a given size and observers indicated which of the two images the hybrid looked more like using the keypad. The *dynamic* condition (with the same 36 trials as Experiment 1) tested the same observers while they viewed the hybrid continuously changing in size, either growing larger or smaller. Importantly, the size changes were linear in distance (arc-tan in diameter), simulating how objects changed size in the world. Observers were told to release the space bar when the image looked equally like both component images (the *point of subjective equality*). The hybrid continued decreasing in size or increasing in size all the way to the end even after observers released the space bar, to eliminate any incentive to end the trials early.

## Experiments 1 and 2 Results

**Figure 2:**
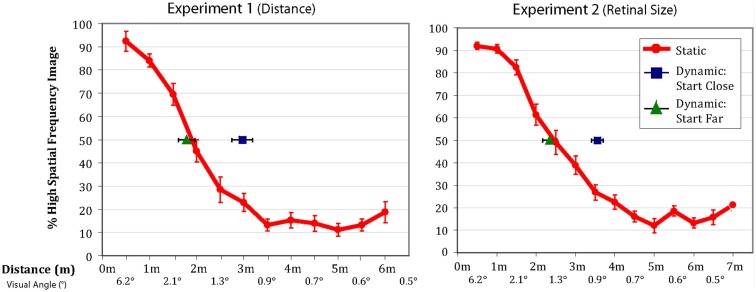
**Data from Experiments 1 and 2**. The red lines indicate the percentage of trials on which observers in the *static* condition reported seeing the high frequency image (error bars are ±1 SEM). The green triangles and blue squares show the distance/visual angle at which observers reported the point of subjective equality in the dynamic conditions (error bars are ±1 SEM). When starting far from the image observers’ reported lined up perfectly with their reported point of subjective equality from the static conditions. When starting close to the image, observers tended to stick with the high frequency image considerably longer than predicted by their static responses.

All observers successfully completed the task. Figure 2 shows that the patterns of results were very similar between the physical distance change and the retinal size change. The red lines represent the percentage of time observers’ reported seeing the high spatial frequency component of the hybrid in the *static* condition. Importantly, the results in the *dynamic* conditions differed significantly depending on whether observers were walking toward or walking away from the image (and similarly, if the image was increasing or decreasing in size).

In the observer-object approaching conditions of the two experiments, the point of subjective equality was nearly identical to the point of subjective equality of the static condition [size of hysteresis effect: Experiment 1 = 11.1 cm, *t*(7) = 0.64, *p* = 0.54; Experiment 2 = 11.6 cm^3^, *t*(17) = 0.76, *p* = 0.45]. However, in the observer-object receding conditions, where observers walked away from the hybrid image, or when the image decreased in size, they demonstrated considerable hysteresis [size of hysteresis effect: Experiment 1 = 106.9cm, *t*(7) = 4.96, *p* = 0.002; Experiment 2 = 109.4cm, *t*(17) = 5.94, *p* = 0.00002], reporting the point of subjective equality further away from the image than when they were viewing the images in their respective static conditions. In other words, they were responding as if they were seeing details that should not have been perceptible. The size of the hysteresis effect is substantial with observers reporting being at the 50% point where they had been at the 23% (Experiment 1) and 24% (Experiment 2) points in the static condition.

It is possible that the lack of hysteresis in the observer-object approaching condition would be caused by a failure to distinguish the two component images when they are presented small or far away. To examine this, we ran an additional experiment (*N* = 4) that was the same as Experiment 2 except we always displayed the two component images at their largest size, even when the hybrid was shown at a smaller size. There is thus no asymmetry in knowledge about the available options in the far case compared to the close case. The results exactly replicated those from Experiment 2, with a hysteresis size of 116.2 cm (SEM: 9.2 cm) in the close condition and 5.3 cm (SEM: 23.6 cm) in the far condition. This suggests that the asymmetric hysteresis is not a function of the available information about the component images.

### Effects of the measurement method

In the static condition, we employed the method of constant stimuli to map out observers’ psychometric function for distinguishing the low and high spatial frequency components of a hybrid. As we varied the distance observers stood from the hybrid, they reported which component image the hybrid most strongly resembled. This method allowed us to obtain an unbiased measure of the entire psychometric function for the relative salience of the two component images, giving us an estimate of the point where the two components of the hybrid are equally likely to be perceived by our observers – a point of subjective equality. However, in the dynamic condition, we by necessity employed a continuous method, similar to the method of limits, by having observers continuously approach or step back from the hybrid and report which component image it most closely resembled (similar to the distance-based threshold measurements of Pelli, 1999). Measurement of thresholds in this manner can be subject to progression effects, in which observers may continue to report a previous stimulus even if their perception changes, which is why many modern psychophysical studies use the method of constant stimuli or a staircasing procedure instead. One might think that such progression effects explain the hysteresis we observe here; however, the asymmetric hysteresis we observe in our data suggests they do not.

The most straightforward reason to believe the hysteresis effect is not a result of the method used to measure it, is the asymmetry we observe between approaching and receding conditions. If the results of the dynamic condition were explained by a simple progression effect, observers should be delayed in reporting the stimulus perception changing both when the face approaches and when it is receding. Instead, observers’ reported point of subjective equality in the “from far” condition is nearly identical to the point derived from the static condition (although the effect observed in the “from far” condition does have a small, several centimeter hysteresis effect, perhaps as a result of progression effects resulting from the method of measurement; see Figure 2). By contrast, the “from close” condition displays an extremely large hysteresis effect, inconsistent with a simple progression effect. This suggests that a progression effect in the dynamic conditions as a result of the method of measurement does not explain the hysteresis we observe.

## Experiment 3 Materials and Methods

**Figure 3:**
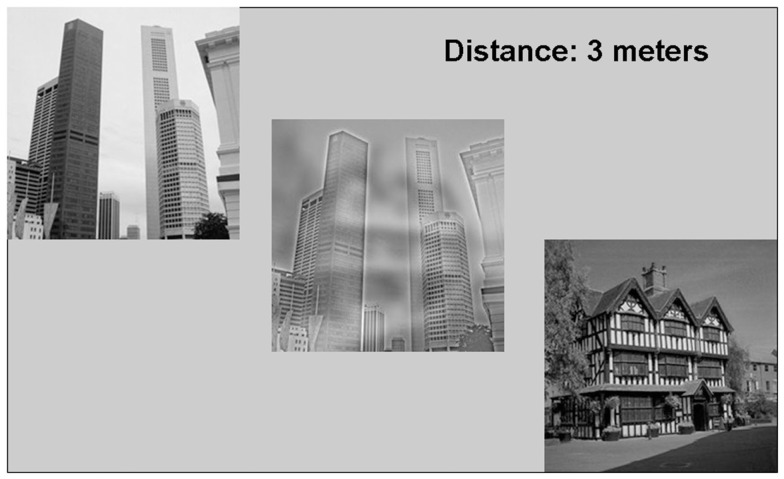
**Example display from Experiment 3**. The images on the left and right of the display are the images composing the hybrid image located in the center of the display. Observers’ task was to stand at the distance indicated and report whether the hybrid looked more like the image on the left or the image on the right.

Faces are sometimes thought to be unique stimuli, processed independently from other objects (e.g., Kanwisher and Yovel, 2009). In fact, a significant literature has formed focusing on how we process faces in particular in the spatial frequency domain. While in general the spatial frequencies used in face processing tasks seems to be highly flexible (Sowden and Schyns, 2006; Pilz et al., 2009), there are some task-related differences in spatial frequency information use in faces. For example, information from different spatial frequencies may be used more for facial identity than emotion (for a review, see Sowden and Schyns, 2006) and this may correspond to two different processing pathways in the brain, one based on the fusiform and one based on the amygdala (Vuilleumier et al., 2003). Are the present hysteresis results unique to the use of face stimuli with a task focused on emotion processing? To examine this question, in Experiment 3 we used images of scenes rather than faces to examine hysteresis (see Figure 3). The task was otherwise the same as Experiment 1.

### Observers

Fourteen observers participated in Experiment 3. They had normal or corrected to normal vision and normal spatial frequency perception, as tested with the Functional Acuity Contrast Test (Vision Sciences Research Corporation, San Ramon, CA, USA). All observers gave informed consent.

### Procedure

All methods were the same as Experiment 1, except that rather than using 18 hybrid faces differing in emotional content, we used 18 scene hybrids, with the low and high spatial frequency images derived from different basic-level categories of scenes (for example, bedrooms, forests, living rooms; see Figure 3).

## Experiment 3 Results

In the observer-approaching condition, the point of subjective equality was nearly identical to the point of subjective equality of the static condition [size of hysteresis effect: 6 cm, *t*(13) = 0.32, *p* = 0.75]. However, in the condition where observers walked away from the hybrid image, they demonstrated considerable hysteresis [size of hysteresis effect: 54 cm, *t*(13) = 3.09, *p* = 0.009], reporting the point of subjective equality further away from the image than when they were viewing the images in the static condition. The hysteresis was once again asymmetric, as the effect was larger in the walking away condition than the walking toward condition [*t*(13) = 2.80, *p* = 0.015].

The hysteresis effect trended toward a smaller size in scenes than faces (Experiment 3 vs. Experiment 1: 54 vs. 107 cm; *p* = 0.06). This likely reflects the greater incongruity between the low and high spatial frequency components in the scene hybrids than the face hybrids. In particular, as noted in the Appendix, the low spatial frequency component of the hybrid is almost always visible. Thus, hybrids change interpretation because the high spatial frequency image provides new, spatially overlapping information on top of the low spatial frequency image. Thus, spatial overlap between the two images is important so that the low spatial frequency image is not visible from close to the image (Oliva et al., 2006). Faces are ideal stimuli for creating such overlap, as faces of the same person necessarily overlap in all but the regions that signify emotional change, and even subtle visual changes can majorly influence the interpretation of the emotion of a face. The scenes are considerably more ambiguous stimuli and thus observers are confronted with a greater degree of incompatible visual information from the low frequency image in the walking away condition. This may cause them to let go of their previous interpretation more quickly.

Nevertheless, the presence of the asymmetric hysteresis effect even with scenes suggests that the present results are a general property of how the visual system processes objects and scenes, rather than a property unique to face processing.

## General Discussion

Whereas the spatial frequency properties of our visual system have been studied extensively (e.g., Blakemore and Campbell, 1969; Graham, 1989), the question of how meaningful information dynamically accumulated across spatial frequencies is integrated into a coherent percept has not received as much attention. Using hybrid faces that present different emotions in low and high spatial frequencies, we observe an asymmetry in the way observers perceive spatial frequency information both over variations in distance (Experiment 1) and retinal size (Experiment 2). We also observe this asymmetry using scene images instead of faces (Experiment 3). In all cases, upon approaching observers act as though they are independently integrating spatial frequency from the image at each moment. However, when the stimulus is receding, observers systematically show a *perceptual hysteresis* effect, holding on to more high spatial frequency details than are perceived in a static stimulus condition.

Interestingly, this asymmetry of spatial frequency integration corresponds to an important fact about the information present in the retinal images as a function of distance: as observers walked forward they gained information in the form of high spatial frequency components and lost very little information (see Appendix). As they walked backward they lost high spatial frequency information from the image (e.g., Loftus and Harley, 2005; Sowden and Schyns, 2006). Thus, under conditions where they are losing information, observers continue to perceive the hybrid using the previous, more detailed information. This is in some sense the optimal thing to do if we wish to make the best inference about what stimuli in the world are causing our current percept (e.g., Helmholtz, 1867; Marr, 1982; Yuille and Kersten, 2006), since we have more information about the image when we are close to it than we do when we are further way. If you see an animal that looks like a tiger, but as you get further away you can no longer see the stripes and so the animal looks like a dog, it is probably not a good idea to suppose the animal has changed into a dog. More likely, the changes results from your losing access to high spatial frequency information. On the other hand, if you see an animal from far away that looks like a dog, but upon getting closer it seems as though it might be a tiger, it is probably best to trust your visual system’s new inference.

### Visual hindsight bias

The hysteresis phenomenon we observe here is related to the visual hindsight bias (Bruner and Potter, 1964; Bernstein et al., 2004; Harley et al., 2004; Sadr and Sinha, 2004), a general phenomenon where observers who know what to expect in a stimulus or a task will change the threshold at which they can detect a stimulus. In other words, observers hold on to previous hypotheses to interpret the present percept. At first, it might be surprising that we do not observe hysteresis in the object approaching condition, where the far distance (or small) object is in a blurry state to start with. Indeed, Bruner and Potter (1964) reported that seeing a very blurry image interfered with the ability to recognize it when it became less blurry. This suggests that observers hold onto the hypotheses they formed about the image even in the face of newer and more useful information.

Importantly, however, such visual hindsight studies are cases where observers do not know what the image might possibly be: a very blurry image is compatible with a huge number of hypotheses and potential perceptual grouping interpretations. Thus, hysteresis observed under such conditions may be a result of either: (1) the visual system erroneously overweighting the hypothesis it started with, even though it should have been discarded when new information became available, or (2) an inability of the visual system to derive any other possible alternative hypothesis when it is already entertaining a particular hypothesis. The current experiment indicates that when observers know which hypotheses are possible (they know which two images make up the hybrid), perceptual hysteresis was not observed as more information became available. This provides strong evidence that observers’ visual systems weight the relevant hypotheses based on how strong the available information is. Thus, the most likely source of failure in visual hindsight studies (Bruner and Potter, 1964; Sadr and Sinha, 2004) is a failure of observers’ to bring to mind alternative hypotheses about what the image might represent, rather than a failure to weight the evidence for such hypotheses (note that this is compatible with results from priming studies reported by Sadr and Sinha, 2004).

### Conclusion

We find that observers stick with their interpretation of hybrid images when those images are getting smaller and/or further away, but not when the images are growing and/or getting closer to the observer. Beyond mere visual identification, these findings speak to a core principle of how our perceptual systems make use of information that evolves over time: observers take into account information from memory that provides additional constraints on the correct interpretation of an image.

## Conflict of Interest Statement

The authors declare that the research was conducted in the absence of any commercial or financial relationships that could be construed as a potential conflict of interest.

## Figures and Tables

**Figure A1:**
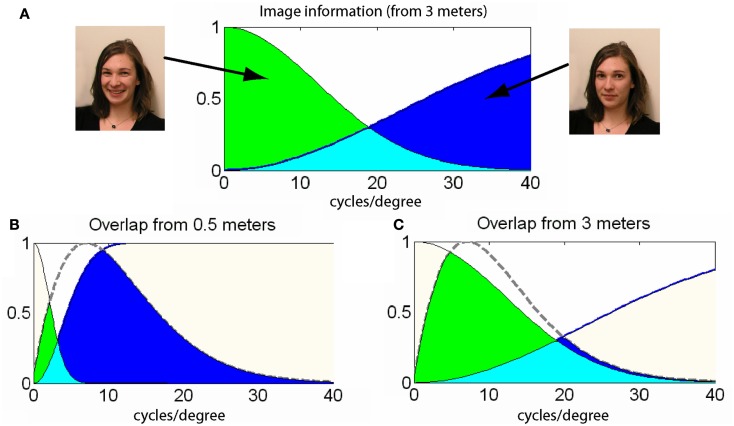
**(A)** The information contained in a hybrid image is a mixture of the information originating from the high spatial frequency component of an image (blue) and the low spatial frequency component of another image (green). **(B)** From a distance of 3 m, the information visible to human observers (the information available from a standard CSF, depicted as the dashed-gray line) is predominantly driven by the low spatial frequency component: observers should preferentially perceive the happy face. **(C)** From a distance of 0.5 m, the visible information is predominately from the high spatial frequency component: as a result, observers should see a neutral face.

**Figure A2:**
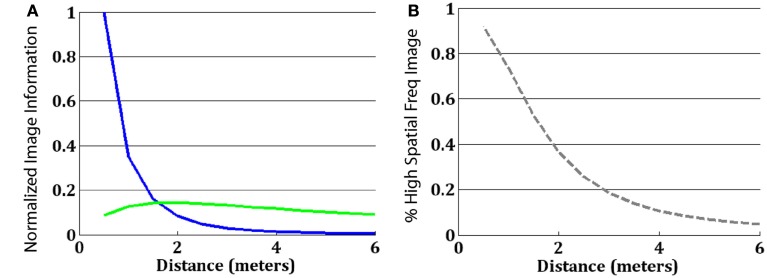
**(A)** The total amount of information available from each component after taking a weighted sum by the CSF at a given distance. The *y*-axis is normalized to put the maximum information at 1.0. **(B)** The percentage of all information about the image available at a given distance that comes from the high spatial frequency component.

## Footnotes

^1^We attempted to make the task easier for observers by having them report three points to the experimenter: when the hybrid became ambiguous – e.g., when the image began to have at least some component of both percepts, rather than being 100% one or the other; when the perceived image was 50% of each component; and when it was no longer ambiguous at all and corresponded entirely to the other component. The distances of these points were marked by the experimenter using a ruler that was taped to the ground. For data analysis, we are only interested in the point of subjective equality, which is the common response between experiments 1 and 2.

^2^The hybrids were resized to be smaller using bilinear interpolation. At the smallest size the hybrids comprised only 62 pixels, meaning that the highest spatial frequencies were filtered by the resizing process and the monitor rather than by observers’ contrast sensitivity function.

^3^We report the results of Experiment 2 in terms of simulated distance, e.g., the distance in Experiment 1 that corresponds to the visual angle from Experiment 2. This allows for easy comparisons across the two experiments. The visual angles are reported in Figure 2.

## Appendix

### Role of the contrast sensitivity function

The human visual system is more sensitive to contrast than to absolute levels of illumination, and furthermore is most sensitive to contrast differences that occur at a particular range of frequencies (roughly between 4 and 20 cycles/visual degree; Robson, 1966). This contrast sensitivity function (CSF) serves as a band-pass filter, effectively making low contrast information at particular spatial frequencies invisible. Because of the asymmetric shape of the human CSF, high spatial frequency signals fall-off more steeply than the low spatial frequency signals as the distance between the observer and the image increases, Can this account for the hysteresis effects we observe in the “receding” conditions?

Figure A1A depicts a caricature of the information contained in the hybrid image from Figure 1 (the high spatial frequency component is colored blue; the low spatial frequency component is colored green), assuming complete “whitening” of the signal to remove the 1/f amplitude spectrum characteristic of natural images (Atick and Redlich, 1992). To create the hybrids, we used Gaussian filters that eliminated the high spatial frequencies of one image and the low spatial frequencies of the other and then summed them together. Thus, from any distance, the information in the hybrid is a mixture of information from the two component images. For a given hybrid mixture, the information perceived, however, depends on the distance of the observer to the image, and their CSF (for implementation details, see Oliva et al., 2006). What happens if we filter the information from the hybrid image with the human CSF? An example of human CSF is depicted as a gray dashed line in Figure A1. Figure A1B depicts what information is available under the CSF from a distance of 3 m from the hybrid. By taking a CSF-weighted sum of the information available in the image, we can see that at this distance, nearly all of the high spatial frequency image (blue) is too high frequency to be seen, as it is filtered by the CSF. Thus, nearly the entire visible part of the hybrid image belongs to the low spatial frequency component. By contrast, panel C shows the same hybrid image filtered by the CSF, for a viewing distance of 0.5 m. Here, the hybrid information is shifted into much higher spatial frequencies (in cycles/degree), and so in addition to the low spatial frequency component information there is now a large amount of information from the high spatial frequency component. We can attempt to predict which image observers will see from a given distance by simply examining what percentage of the information available after filtering by the CSF belongs to the high spatial frequency component vs. the low spatial frequency component. Figure A2 depicts this information. Panel A shows, as a function of distance, how much information about the high spatial frequency image (blue) is available after filtering by the CSF, and how much information about the low spatial frequency component (green) is available (taking a weighted sum of the blue and green lines from Figure A1A, using the CSF at the particular distance as the weights). As found by Loftus and Harley (2005), we find that for this size stimulus across this range of distances, the CSF effectively acts as a low-pass filter. That is, information about the low spatial frequency component is nearly always available to the viewer, whereas information about the high spatial frequency component becomes available only when observers are close to the image. In this way, hybrid images are more like an image in which the detailed high spatial frequency image masks the still-visible blurry image than an image in which observers transition completely from having information about one image to having information about another (for details, see Oliva et al., 2006).

Figure A2B is a simple ratio of the blue line in Panel A to the sum of both lines, showing what proportion of the total information about either component that is available to observers at a given distance comes from the high spatial frequency image. Comparing this with the psychometric function of observers from Figure 2 demonstrates a nice fit between the relative amount of information available to observers from a particular component and the likelihood the observers report perceiving that component. Thus, in the static condition observers appear to behave exactly as we would expect from their CSF, reporting that they perceive the component from which they are receiving the most information.

In the dynamic condition, observers’ in the “from far” condition act exactly as we would expect from both the CSF and, empirically, from their psychometric function in the static condition. When the high spatial frequency component image accounts for more than 50% of the total information, they report beginning to perceive that image more than the low spatial frequency component.

However, in the “from close” condition, observers do not report changing their interpretation of the image’s appearance until much later (at a point where they report approximately 23% high spatial frequency in the static condition). This phenomenon is clearly not predictable by the CSF alone, which predicts the exact same point of equality between the images as in both the static and “from far” conditions – the point where more than 50% of the image information comes from the low spatial frequency image. This suggests that observers are taking into account their previous perception of the image in deciding what the image looks like (e.g., how to perceptually organize it), and, importantly, doing so only when they are losing information about the image.

## References

[B1] Atick J. J. Redlich A. N. (1992). What does the retina know about natural scenes? Neural Comput. 4, 196–210 10.1162/neco.1992.4.2.196

[B2] Bar M. (2004). Visual objects in context. Nat. Rev. Neurosci. 5, 617–629 10.1038/nrn1476 15263892

[B3] Bar M. Kassam K. S. Ghuman A. S. Boshyan J. Schmidt A. M. Dale A. M. (2006). Top-down facilitation of visual recognition. Proc. Natl. Acad. Sci. U.S.A. 103, 449–454 10.1073/pnas.0507062103 16407167 PMC1326160

[B4] Bernstein D. M. Atance C. Loftus G. R. Meltzoff A. N. (2004). We saw it all along: visual hindsight bias in children and adults. Psychol. Sci. 15, 264–267 10.1111/j.0963-7214.2004.00663.x 15043645 PMC3640979

[B5] Blakemore C. Campbell F. W. (1969). On the existence of neurons in the human visual system selectively sensitive to the orientation and size of retinal images. J. Physiol. (Lond.) 203, 237–260 5821879 10.1113/jphysiol.1969.sp008862 PMC1351526

[B6] Bruner J. S. Potter M. C. (1964). Interference in visual recognition. Science 144, 424–425 10.1126/science.144.3617.424 14169336

[B7] Graham N. (1989). Visual Pattern Analyzers. New York: Oxford

[B8] Harley E. M. Carlsen K. A. Loftus G. R. (2004). The “Saw-it-all-along” effect: demonstrations of visual hindsight bias. J. Exp. Psychol. Learn Mem. Cogn. 30, 960–968 10.1037/0278-7393.30.5.960 15355129

[B9] Helmholtz H. (1867). Handbuch der Physiologischen Optik. Leipzig: Voss

[B10] Kanwisher N. Yovel G. (2009). “Cortical Specialization for Face Perception in Humans,” in Handbook of Neuroscience for the Behavioral Sciences, eds Cacioppo J. T. Berntson G. G. (Hoboken: John Wiley and Sons).

[B11] Kihara K. Takeda Y. (2010). Time course of the integration of spatial frequency-based information in natural scenes. Vision Res. 50, 2158–2162 10.1016/j.visres.2010.08.012 20723557

[B12] Kubovy M. Pomerantz J. R. (1981). Perceptual Organization. Hillsdale: Lawrence Erlbaum

[B13] Lindeberg T. (1994). Scale-Space Theory in Computer Vision. Boston: Kluwer

[B14] Loftus G. R. Harley E. M. (2004). How different spatial-frequency components contribute to visual information acquisition. J. Exp. Psychol. Hum. Percept. Perform. 30, 104–118 10.1037/0096-1523.30.1.104 14769071

[B15] Loftus G. R. Harley E. M. (2005). Why is it easier to recognize someone close than far away? Psychon. Bull. Rev. 12, 43–65 10.3758/BF03196348 15948283

[B16] Maier J. X. Neuhoff J. G. Logothetis N. K. Ghazanfar A. A. (2004). Multisensory integration of looming signals by rhesus monkeys. Neuron 43, 177–181 10.1016/j.neuron.2004.06.027 15260954

[B17] Marr D. (1982). Vision. San Francisco: Freeman

[B18] Nakayama K. He Z. J. Shimojo S. (1995). “Visual Surface Representation: A Critical Link Between Lower-Level and Higher Level Vision. Vision,” in Invitation to Cognitive Science, eds Kosslyn S. M. Osherson D. N. (Cambridge: MIT Press), 1–70

[B19] Oliva A. Schyns P. G. (1997). Coarse blobs or fine edges? Evidence that information diagnosticity changes the perception of complex visual stimuli. Cogn. Psychol. 34, 72–107 10.1006/cogp.1997.0667 9325010

[B20] Oliva A. Torralba A. Schyns P. G. (2006). Hybrid images. ACM transactions on graphics. ACM Siggraph 25, 527–532 10.1145/1141911.1141919

[B21] Palmer S. E. (1999). Vision Science: Photons to Phenomenology. Cambridge: MIT Press

[B22] Peli E. (1990). Contrast in complex images. J. Opt. Soc. Am. A 7, 2032–2040 10.1364/JOSAA.7.002032 2231113

[B23] Pelli D. G. (1999). Close encounters – an artist shows that size affects shape. Science 285, 844–846 10.1126/science.285.5429.844 10454935

[B24] Pilz K. Bülthoff H. Vuong Q. (2009). Learning influences the encoding of static and dynamic faces and their recognition across different spatial frequencies. Vis. Cogn. 17, 716–735 10.1080/13506280802340588

[B25] Pilz K. S. Vuong Q. C. Bülthoff H. Thornton I. M. (2011). Walk this way: approaching bodies can influence the processing of faces. Cognition 118, 17–31 10.1016/j.cognition.2010.09.004 21047624

[B26] Robson J. G. (1966). Spatial and temporal contrast sensitivity functions of the visual system. J. Opt. Soc. Am. 56, 1141–1142 10.1364/JOSA.56.001141

[B27] Rovamo J. Franssila R. Näsänen R. (1992). Contrast sensitivity as a function of spatial frequency, viewing distance and eccentricity with and without spatial noise. Vis. Res. 32, 631–637 10.1016/0042-6989(92)90179-M 1413547

[B28] Sadr J. Sinha P. (2004). Object recognition and random image structure evolution. Cogn. Sci. 28, 259–287 10.1207/s15516709cog2802_7

[B29] Schyns P. G. Oliva A. (1994). From blobs to boundary edges: evidence for time- and spatial-scale-dependent scene recognition. Psychol. Sci. 5, 195–200 10.1111/j.1467-9280.1994.tb00500.x

[B30] Schyns P. G. Oliva A. (1999). Dr. Angry and Mr. Smile: when categorization flexibly modifies the perception of faces in rapid visual presentations. Cognition 69, 243–265 10.1016/S0010-0277(98)00069-9 10193048

[B31] Smith F. W. Schyns P. G. (2009). Smile through your fear and sadness: transmitting and identifying facial expression signals over a range of viewing distances. Psychol. Sci. 20, 1202–1208 10.1111/j.1467-9280.2009.02427.x 19694983

[B32] Sowden P. T. Schyns P. G. (2006). Channel surfing in the visual brain. Trends Cogn. Sci. (Regul. Ed.) 10, 538–545 10.1016/j.tics.2006.10.007 17071128

[B33] Vuilleumier P. Armony J. L. Driver J. Dolan R. J. (2003). Distinct spatial frequency sensitivities for processing faces and emotional expressions. Nat. Neurosci. 6, 624–631 10.1038/nn1057 12740580

[B34] Yuille A. Kersten D. (2006). Vision as Bayesian inference: analysis by synthesis? Trends Cogn. Sci. (Regul. Ed.) 10, 301–308 10.1016/j.tics.2006.05.002 16784882

